# PERFORMANCE ON THE MOCA IS NOT ASSOCIATED WITH COGNITIVE RESERVE IN COMMUNITY-DWELLING OLDER ADULTS

**DOI:** 10.1093/geroni/igae098.4043

**Published:** 2024-12-31

**Authors:** Marcella Barneclo, A Lisette Isenberg, Joy Stradford, Kimberly Espejo, Judy Pa

**Affiliations:** University of California, San Diego, San Diego, California, United States; University of California, San Diego, La Jolla, California, United States; University of California San Diego, La Jolla, California, United States; UC San Diego, San Diego, California, United States; University of California, San Diego, La Jolla, California, United States

## Abstract

The Cognitive Reserve (CR) hypothesis suggests that more experiential resources (e.g. education, occupation, recreational activities) can help individuals combat age-related cognitive decline and maintain higher cognitive function with age. As such, higher CR has been implicated in greater cognitive resilience potentially correlated to delaying the onset and progression of Alzheimer’s Disease (AD). Previous research in cognitively normal populations found that the Montreal Cognitive Assessment (MoCA), a well-established neuropsychological assessment of global cognition used to identify Mild Cognitive Impairment (MCI) or mild AD in older adults, may reflect CR. However, limited information exists on the association between MoCA and CR for MCI. The present study investigates the relationship between MoCA and CR in older adults with early MCI using the Cognitive Reserve Index questionnaire (CRIq), a proxy measure of CR determined by education, occupational activity, and recreational activity. The study includes 138 older adults aged 50-85 with early MCI, comprising 50 males and 88 females, with an average age of 66.1 ± 7.40. No correlation between MoCA and total CRIq scores (r = 0.12, p = 0.17), or the subcategories of working activity (r = 0.08, p = 0.35), education (r = 0.05, p = 0.54), and leisure time (r = 0.11, p = 0.19) were observed. Age was included as a covariate in the models. There were no differences between sexes for CRIq scores, MoCA scores, or years of education, respectively. These findings suggest that performance on the MoCA is not associated with CR in older adults with early MCI.

